# ID 158 - Oportunidades de Tratamento para Indivíduos com Hiperoxalúria Tipo 1

**DOI:** 10.5327/2237-9622.2025.v34s1.158

**Published:** 2025-11-25

**Authors:** Ternize Mariana Guenkka, Kelli Carneiro de Freitas Nakata, Helder Cassio de Oliveira, Luci Emília Grzybowski de Oliveira, Priscilla Perez da Silva Pereira

**Keywords:** monitoramento do horizonte tecnológico, doenças raras, hiperoxalúria, lumasirana

## Abstract

**Introdução:**

Hiperoxalúria primária é uma doença metabólica rara causada por mutações genéticas que afetam o metabolismo do glioxilato, resultando em produção excessiva de oxalato e consequentemente formação de cálculos renais, nefrocalcinose, podendo levar à redução da função renal e oxalose sistêmica. A lumasirana é o primeiro e único tratamento específico para a doença. Trata-se de um ácido ribonucleico RNA terapêutico de interferência que inibe a síntese de oxalato.

**Método:**

Monitoramento do horizonte tecnológico com buscas nas bases MEDLINE, Embase, LILACS, Cochrane Library, Clinical Trials, Cortellis, agências internacionais de avaliação de tecnologias em saúde. Os critérios de inclusão foram: ensaios clínicos a partir da fase 2; estudos que avaliassem segurança, eficácia e/ou eventos adversos do lumasirana. Foram elegíveis estudos publicados ou não, em texto completo ou resumo, sem restrição de idioma. O processo de leitura e extração dos dados foi realizada por um revisor e confirmada por um segundo.

**Resultados:**

Foram encontrados 165 títulos, após avaliação de duplicações e critérios de elegibilidade foram analisados 12 artigos referentes a quatro estudos: 1) ILLUMINATE-A (n=39, quatro publicações); 2) ILLUMINATE-B (n=18, duas publicações); 3) ILLUMINATE-C (n=21, três publicação); 4) um estudo de fase 2 (n=21, duas publicações). Tratam-se de estudos multicêntricos, dois deles ainda não finalizados. O estudo ILLUMINATE-A demonstrou decréscimo da média de excreção urinária de oxalato em 24 horas, da linha de base até seis meses, de 11,8% (EP 3,8) no grupo placebo e 65,4% (EP 2,9) no grupo lumasirana. Na fase de extensão, a redução foi mantida no grupo lumasirana (64,1%, 58,1% e 63% em 12, 24 e 36 meses, respectivamente). A população de lactentes e crianças <6 anos (ILLUMINATE-B) também experimentou redução média percentual para esse desfecho na ordem de -68,41% (SE 5,6) em seis meses e -63,23% (SE 7,23) em 12 meses, comparado a linha de base. Em indivíduos com doença renal avançada, subgrupo sem hemodiálise, a variação percentual foi de -10,6% ± 6,8 (IC 95% −32,0 a 10,9) no mês 6. Lumasirana promoveu ainda decréscimo no percentual da relação oxalato urinário/creatinina (24h); no oxalato plasmático e na taxa de eventos de cálculo renal, além de melhorar ou estabilizar a nefrocalcinose. Os indivíduos que receberam lumasirana apresentaram eventos adversos com gravidade ligeira ou moderada. Os eventos mais frequentes foram: reação no local da injeção; dor de cabeça; dor abdominal; infecção do trato respiratório superior; pirexia; vômito e diarreia. As agências de ATS NICE () e CADTH () emitiram parecer favorável ao uso da medicação para o tratamento de hiperoxalúria tipo 1.

**Conclusão:**

Embora o tamanho amostral desses estudos seja pequeno e não esteja disponível dados de longo prazo, há de se considerar que a doença é rara, e que a lumasirana assiste a uma necessidade médica não atendida tendo sido bem tolerado por lactentes, crianças, adultos e indivíduos com comprometimento renal. O preço de unidade de lumasirana é significativo para um tratamento em longo prazo, entretanto, por ser uma doença rara, é esperado um número pequeno de pacientes. Ainda assim, para determinar a eficiência do tratamento com lumasirana é necessário um estudo de custo-efetividade da tecnologia em relação ao padrão de tratamento atual.

